# Identification of recent cases of hepatitis C virus infection using physical-chemical properties of hypervariable region 1 and a radial basis function neural network classifier

**DOI:** 10.1186/s12864-017-4269-2

**Published:** 2017-12-06

**Authors:** James Lara, Mahder Teka, Yury Khudyakov

**Affiliations:** Division of Viral Hepatitis, National Center for HIV, Hepatitis, TB and STD Prevention, Centers for Disease Control and Prevention, Atlanta, GA 30333 USA

## Abstract

**Background:**

Identification of acute or recent hepatitis C virus (HCV) infections is important for detecting outbreaks and devising timely public health interventions for interruption of transmission. Epidemiological investigations and chemistry-based laboratory tests are 2 main approaches that are available for identification of acute HCV infection. However, owing to complexity, both approaches are not efficient. Here, we describe a new sequence alignment-free method to discriminate between recent (R) and chronic (C) HCV infection using next-generation sequencing (NGS) data derived from the HCV hypervariable region 1 (HVR1).

**Results:**

Using dinucleotide auto correlation (DAC), we identified physical-chemical (PhyChem) features of HVR1 variants. Significant (*p* < 9.58 × 10^−4^) differences in the means and frequency distributions of PhyChem features were found between HVR1 variants sampled from patients with recent vs chronic (R/C) infection. Moreover, the R-associated variants were found to occupy distinct and discrete PhyChem spaces. A radial basis function neural network classifier trained on the PhyChem features of intra-host HVR1 variants accurately classified R/C-HVR1 variants (classification accuracy (CA) = 94.85%; area under the ROC curve, AUROC = 0.979), in 10-fold cross-validation). The classifier was accurate in assigning individual HVR1 variants to R/C-classes in the testing set (CA = 84.15%; AUROC = 0.912) and in detection of infection duration (R/C-class) in patients (CA = 88.45%). Statistical tests and evaluation of the classifier on randomly-labeled datasets indicate that classifiers’ CA is robust (*p* < 0.001) and unlikely due to random correlations (CA = 59.04% and AUROC = 0.50).

**Conclusions:**

The PhyChem features of intra-host HVR1 variants are strongly associated with the duration of HCV infection. Application of the PhyChem biomarkers to models for detection of the R/C-state of HCV infection in patients offers a new opportunity for detection of outbreaks and for molecular surveillance. The method will be available at https://webappx.cdc.gov/GHOST/ to the authenticated users of Global Hepatitis Outbreak and Surveillance Technology (GHOST) for further testing and validation.

## Background

Hepatitis C is a liver inflammation caused by HCV. Approximately 80% of HCV-infected individuals develop a life-long (chronic) infection, while the other experience a short-term infection and clear the virus [1]. Accurate identification of acute or recent hepatitis C infection is essential for identification of outbreaks and for devising timely public health interventions to interrupt transmissions. In outbreak settings, epidemiological investigation allows for the detection of recent infection. In surveillance settings, however, epidemiological support may be limited, and information on duration of HCV infection may not be available. Recent infection can be also identified by detection of HCV seroconversion and/or by gauging anti-HCV IgG avidity [2, 3]. However, detection of seroconversion is time-consuming, and avidity tests are not broadly available, thus rendering both approaches of impractical for surveillance. To date, there are not cost-effective and reliable methods suitable for large-scale identification of recently acquired HCV infection.

We have recently shown that genetic diversity of intra-host HVR1 variants is associated with duration of HCV infection and can be applied for the detection of recent (R) or chronic (C) infections [4]. The study showed that the R/C state of infection correlated with position-specific amino-acid PhyChem properties in HVR1. However, methods that utilize sequence-specific features require multiple sequence alignment (MSA), which can be an NP-complete problem [5] or computationally expensive, especially when applied to the next-generation sequencing (NGS) data. In addition, extraction and identification of high-quality biomarkers from nucleotide sequences, beyond sequence patterns and population diversity, are not trivial and remain largely unexplored.

There are myriads of ways for transforming DNA/RNA sequence data into numerical representations. One of the most informative representations is based on using scads of PhyChem properties for individual nucleotides or various combinations of nucleotides [6]. The aim of this study was two-fold: firstly, to investigate DNA data transformation techniques for identifying the PhyChem features of HVR1 variants from unaligned sequences; and, secondly, to evaluate the identified features for the accurate detection of the R/C states of HCV infection. Here, we investigated applicability of the HVR1 NGS data for the differential assessment of duration of HCV infection. We describe the application of the DNA dinucleotide-based auto-covariance (DAC) method to effectively identify relevant PhyChem features of HVR1 variants, and the implementation of a radial basis function neural network (RBFNN) classifier to discriminate between R- and C-associated intra-host HVR1 variants without need of MSA prior to the classification test. We also discuss the use of this approach in the domain of cyber-molecular technology for rapid detection of the R/C state of HCV infection in surveillance settings.

## Methods

### HVR1 sequence data

Sequences of the intra-host HVR1 variants (*n* = 15,041) sampled from 301 HCV-infected patients diagnosed with chronic (*n* = 123) or recent (*n* = 178) infection –patients infected for more than 1 year or less than a year, respectively– were described in our previous study [4]. The four nucleotide (nt) bases (A, G, U and C) present in the HVR1 of HCV RNA genomes were converted to the corresponding DNA format (A, G, T and C) because of the greater availability of PhyChem properties for the DNA-specific base T than for the RNA-specific U.

For statistical and classification tests, the data were divided into two datasets (training/testing). Sequences of intra-host HVR1 variants (*n* = 5681) derived from 222 persons (R, *n* = 124; C, *n* = 98) were used for training of the classifier, while remainder of the data (*n* = 9360) from 79 persons (R, *n* = 54; C, *n* = 25) were used for testing of the classifier. HVR1 variants comprising the training and test datasets were represented as feature vectors of 148 PhyChem indexes and assigned to the R or C class based on the R/C infection status of the corresponding patient.

To examine effects of data randomization on performance of RBFNN classifier, five training datasets were generated from the HVR1 sequence data, where instances in each dataset were randomly shuffled using different randomization seeds. In addition, to account for the possibility of random correlations in data, four random datasets were generated from the training dataset, each generated by randomly class-labeling the instances using different randomization seeds.

### PhyChem features

The PhyChem indices of DNA nt dimers used to generate feature vectors representing the PhyChem features of HVR1 variants were derived from [6, 7]. Correlation measures for the same PhyChem index between two nt dimers separated by a distance (*Lag*) along the sequence were calculated using the following equation (described in [8]):

$$ \mathit{\mathsf{DAC}}\left(\mathit{\mathsf{u}},\mathit{\mathsf{L}\mathsf{ag}}\right)=\sum \limits_{\mathit{\mathsf{i}}=\mathsf{1}}^{\mathit{\mathsf{L}}-\mathit{\mathsf{L}\mathsf{ag}}-\mathsf{1}}\left({\mathit{\mathsf{P}}}_{\mathit{\mathsf{u}}}\ \left({\mathit{\mathsf{R}}}_{\mathit{\mathsf{i}}}{\mathit{\mathsf{R}}}_{\mathit{\mathsf{i}}+\mathsf{1}}\right)-{\overline{\mathit{\mathsf{P}}}}_{\mathit{\mathsf{u}}}\right)\left({\mathit{\mathsf{P}}}_{\mathit{\mathsf{u}}}\ \left({\mathit{\mathsf{R}}}_{\mathit{\mathsf{i}}+\mathit{\mathsf{L}\mathsf{ag}}}{\mathit{\mathsf{R}}}_{\mathit{\mathsf{i}}+\mathit{\mathsf{L}\mathsf{ag}}+\mathsf{1}}\right)-{\overline{\mathit{\mathsf{P}}}}_{\mathit{\mathsf{u}}}\right)/\left(\mathit{\mathsf{L}}-\mathit{\mathsf{L}\mathsf{ag}}-\mathsf{1}\right) $$

where *u* is a PhyChem index, *L* is the length of the HVR1 sequence, (*R*_*i*_ *R*_*i* + 1_) term is the numerical value of PhyChem index *u* for the Nt dimer *R*_*i*_ *R*_*i* + 1_ at position *i*, and ${\overline{P}}_u$ is the average value of the PhyChem index *u* along the HVR1 sequence, which is calculated as follows:

$$ {\overline{\mathit{\mathsf{P}}}}_{\mathit{\mathsf{u}}}=\sum \limits_{\mathit{\mathsf{j}}=\mathsf{1}}^{\mathit{\mathsf{L}}-\mathsf{1}}{\mathit{\mathsf{P}}}_{\mathit{\mathsf{u}}}\ \left({\mathit{\mathsf{R}}}_{\mathit{\mathsf{j}}}{\mathit{\mathsf{R}}}_{\mathit{\mathsf{j}}+\mathsf{1}}\right)/\left(\mathit{\mathsf{L}}-\mathsf{1}\right) $$

Calculations were performed as implemented in the Pse-in-One software (v1.0.3, 2015–08-21 dev) [8], and done in a manner so that length of the PhyChem feature vector is N*Lag, where *N* is the number of DNA PhyChem indices (*N* = 148) and *Lag* = 1.

### Comparative analysis of the HVR1 PhyChem variants

The HVR1 PhyChem variants derived from sequences of intra-host HVR1 variants from chronically infected patients were compared with PhyChem profiles of variants derived from recently infected patients. We examined the differences between the population means for a given PhyChem index of HVR1 variants sampled from acute and chronic patients. To illustrate differences in binned plots, values for the same PhyChem index between two contiguous nt dimers were binned into equal-width bins (threshold = 0.006). Statistical analysis of differences in means of nt frequencies between the R/C patient-derived HVR1 variants were also conducted. In addition, differences in the PhyChem properties between HVR1 PhyChem variants were examined by the multi-dimensional scaling (MDS) technique as implement in [9]. Briefly, the MDS algorithm iteratively moves the points around in a kind of simulation of a physical model, where there is a force pushing them apart or together. A Euclidean distance matrix was computed to represent the spacing of the HVR1 PhyChem variants comprising the training dataset in Euclidean space. The two-dimensional MDS projection was initialized by randomizing the positions of the instances (or points). Sammon stress [10] was used as the stress function to define how the difference between the desired and the actual distance between points translates into the forces acting on the points.

### RBFNN classifier and classification schemes

#### RBFNN classifier model

A machine-learning approach based on feed-forward neural networks (FFNNs) was used to examine the practical significance of DAC-based PhyChem features generated from sequences of HVR1 variants for developing computer applications for the R/C assessment. We implemented the Gaussian RBFNN classifier technique as described in [11]. Briefly, the RBFNN is a type of FFNN that uses a Gaussian radial basis function and consists of units divided into three layers: an input layer, a hidden (or radial basis) layer and an output layer (the linear model). The hidden layer of such types of networks are commonly trained using unsupervised learning by k-means clustering and the output layer using supervised learning by logistic regression (for classification tasks) or by linear regression (for regression tasks). For either task, penalized squared error, using a quadratic penalty on the non-bias weights in the output layer, is used as the loss function to find the model’s parameters.

The constructed RBFNN classifier had 2 output units (one output unit per class of infection durations), and the learned model for the *l*th output unit (i.e., class value) is described by the follow formula:

$$ {f}_l\left({x}_1,{x}_2,,\dots, {x}_m\right)=g\left({w}_{l,0}+\sum \limits_{i=1}^b{w}_{l,i}\exp \left(-\sum \limits_{j=1}^m\frac{a_j^2\ {\left({x}_j-{c}_{i,j}\right)}^2}{2{\sigma}_{i,j}^2}\right)\right) $$

where *x*_1_, *x*_2_, …, *x*_*m*_ is the feature vector for the HVR1 PhyChem variant concerned, the activation function *g*(.) is the logistic function, *b* is the number of basis functions, *w*_*i*_ is the weight for each basis function, ${a}_j^2$ is the weight of the *j*th feature, and *c*_*i*, *j*_ and ${\sigma}_{i,j}^2$ are the basis function centers and variances, respectively.

Settings for the parameters *w*_(*l*, )*i*_, ${a}_j^2$, *c*_*i*, *j*_ and ${\sigma}_{i,j}^2$ were established by finding a local minimum of the penalized squared error on the training dataset using the following error function:

$$ {L}_{SSE}=\left(\frac{1}{2}\sum \limits_{i=1}^n\sum \limits_{l=1}^k{\left({y}_{i,l}-{f}_l\left({\overrightarrow{x}}_i\ \right)\right)}^2\right)+\left(\lambda \sum \limits_{l=1}^k\sum \limits_{i=1}^b{w}_{l,i}^2\right) $$

where *k* classes = 2, *y*_*i*_ is the class value for training instance ${\overrightarrow{x}}_i$, the first sum ranges over all *n* instances in the training dataset and *λ* is the ridge parameter establishing the size of the penalty on the weights to control overfitting.

A value setting of 39 that was used for the *b* parameter, which was determined empirically based on the well-known strategy of grid search with cross-validation (GridSearchCV). The hidden unit centers and variances were initialized as follows: the k-means implementation in [12] was used to initialize the *c*_*i*, *j*_, where the number of k clusters was set at 39 and the minimum standard deviation for the clusters set at 1 × 10^−3^; and the initial value of all variance parameters ${\sigma}_{i,j}^2$ in the network was set to the maximum squared Euclidean distance between any pair of cluster centers to prevent initial value of the variance parameters from being too small [11]. The parameter *λ* for the logistic regression was set at 1 × 10^−8^.

#### Tuning of the b parameter

The number of basis functions (i.e., number of hidden units) that are employed in RBF networks is a relevant parameter that requires particular attention as it directly impacts complexity of the model. The GridSearchCV method was used to search through the hyper-parameter space for the best value for parameter *b*. Briefly, GridSearchCV implements a fit and a score method to optimize parameters of a model by cross-validated grid-search over a parameter grid (i.e., a range of values). The lower boundary of the grid was set at 2 and the upper boundary limit was set at 66, which was inferred by clustering the training dataset using an expectation-maximization (EM) algorithm (discussed in [12]). The GridSearchCV implementation used here is as follows: the initial grid is worked on with 2-fold cross-validation (2× CV) to determine the values of parameter *b* based on an evaluation metric(s) (hereafter, classification accuracy). The best point in the grid is then taken and 10× CV is performed with the adjacent point. If a better point is found, then this will act as new center and another 10× CV is performed. This process is repeated until no better point is found or the best (optimal) point is on the border of the grid.

#### Classification schemes

The RBFNN classifier was trained and evaluated on the training dataset comprising PhyChem variants of HVR1 labeled according to the actual R/C class associations, and with the randomly-labeled datasets where class-labels were randomly assigned to the variants. Classification performances of the RBFNN classifier derived from each scheme was also evaluated on the other dataset (i.e., unseen data).

### Applied statistical tests

The Welch two sample t-test was used to examine the statistical significance of differences between the population means in HVR1 variants sampled from acute (*n* = 124) and chronic (*n* = 98) patients. The null hypothesis is that the difference between the means is 0 (making the difference between these two groups not statistically significant) and the alternative hypothesis is that their difference is not zero. The variance parameter was set to ‘false’ to account for the difference in sample size.

The strength of the association of DNA nt’s and PhyChem variables to the R/C durations of infection was measured using the Pearson product-moment correlation coefficient (r). Additionally, the heuristic Merit metric [13] was used to measure the importance of different subsets of DNA PhyChem features for establishing the association between HVR1 and R/C states. Merit scores for various subsets of PhyChem variables were computed using the following formula,

$$ {\mathit{\mathsf{Merit}}}_{\mathit{\mathsf{S}}}=\frac{\mathit{\mathsf{k}}\times \overline{{\mathit{\mathsf{r}}}_{\mathit{\mathsf{ca}}}}}{\sqrt{\mathit{\mathsf{k}}+\left(\mathit{\mathsf{k}}-\mathsf{1}\right)\times \overline{{\mathit{\mathsf{r}}}_{\mathit{\mathsf{aa}}}}}} $$

where $\overline{r_{ca}}$ is the average feature-class correlation and $\overline{r_{aa}}$ is the average feature-feature inter-correlation in a feature subset *S* containing *k* features.

Measures of statistical significance of the pairwise comparisons of classifying schemes performed in this study was done using the corrected resampled two-tailed T-test [14]. The Welch two sample t-test and Pearson product-moment correlation coefficient (r) were implemented in R (v3.0.1). Computations of the corrected two-tailed T-test and of the Merit scores were implemented as discussed in [12].

### Classifier performance evaluation

Four metrics used to evaluate the RBFNN classifier(s) are reported herein: classification accuracy (CA), F_1_ measure, the Mathews correlation coefficient (MCC) and the Receiver Operating Characteristic (ROC) curve, which was summarized as a single value by computing the area of the convex shape below the ROC curve (AUROC). These metrics were computed as follows:

$$ \mathit{\mathsf{CA}}=\frac{\mathit{\mathsf{TP}}+\mathit{\mathsf{TN}}}{\mathit{\mathsf{TP}}+\mathit{\mathsf{TN}}+\mathit{\mathsf{FP}}+\mathit{\mathsf{FN}}}\times \mathsf{100}\% $$

$$ {\mathit{\mathsf{F}}}_{\mathsf{1}}=\mathsf{2}\bullet \left(\frac{\left(\frac{\mathit{\mathsf{TP}}}{\mathit{\mathsf{TP}}+\mathit{\mathsf{F}\mathsf{P}}}\right)\times \left(\frac{\mathit{\mathsf{TP}}}{\mathit{\mathsf{TP}}+\mathit{\mathsf{F}\mathsf{N}}}\right)}{\left(\frac{\mathit{\mathsf{TP}}}{\mathit{\mathsf{TP}}+\mathit{\mathsf{F}\mathsf{P}}}\right)+\left(\frac{\mathit{\mathsf{TP}}}{\mathit{\mathsf{TP}}+\mathit{\mathsf{F}\mathsf{N}}}\right)}\right) $$

$$ \mathit{\mathsf{MCC}}=\frac{\mathit{\mathsf{TP}}\times \mathit{\mathsf{TN}}-\mathit{\mathsf{FP}}\times \mathit{\mathsf{FN}}}{\sqrt{\left(\mathit{\mathsf{TP}}+\mathit{\mathsf{FP}}\right)\left(\mathit{\mathsf{TP}}+\mathit{\mathsf{FN}}\right)\left(\mathit{\mathsf{TN}}+\mathit{\mathsf{FP}}\right)\left(\mathit{\mathsf{TN}}+\mathit{\mathsf{FN}}\right)}} $$

where *TP* is the number of true positives; *TN*, the number of true negatives; *FP*, the number of false positive and *FN*, the number of false negatives. The interpolated curve (TPR vs FPR), made of points whose coordinates are functions of the *threshold* = *θ* ∈ [0, 1], was generated using the following equations:

$$ {\mathit{\mathsf{ROC}}}_{\mathit{\mathsf{x}}}\left(\mathit{\mathsf{\theta}}\right)=\mathit{\mathsf{FP}\mathsf{R}}\left(\mathit{\mathsf{\theta}}\right)=\frac{\mathit{\mathsf{FP}}\left(\mathit{\mathsf{\theta}}\right)}{\left(\mathit{\mathsf{FP}}\left(\mathit{\mathsf{\theta}}\right)+\mathit{\mathsf{TN}}\left(\mathit{\mathsf{\theta}}\right)\right)} $$

$$ {\mathit{\mathsf{ROC}}}_{\mathit{\mathsf{y}}}\left(\mathit{\mathsf{\theta}}\right)=\mathit{\mathsf{TP}\mathsf{R}}\left(\mathit{\mathsf{\theta}}\right)=\frac{\mathit{\mathsf{TP}}\left(\mathit{\mathsf{\theta}}\right)}{\left(\mathit{\mathsf{FN}}\left(\mathit{\mathsf{\theta}}\right)+\mathit{\mathsf{TP}}\left(\mathit{\mathsf{\theta}}\right)\right)} $$

where *FPR* is the false positive rate and *TPR* is the true positive rate. Computation of AUROC values was done by computing the probability that the RBFNN classifier ranks a randomly chosen positive instance above a randomly chosen negative instance, which was accomplished by calculating the *ρ* statistic from the *U* statistic. Equations and description of the method can be found in [12].

## Results

### HVR1 PhyChem features specifically associated with R/C states

**Fig. 1:**
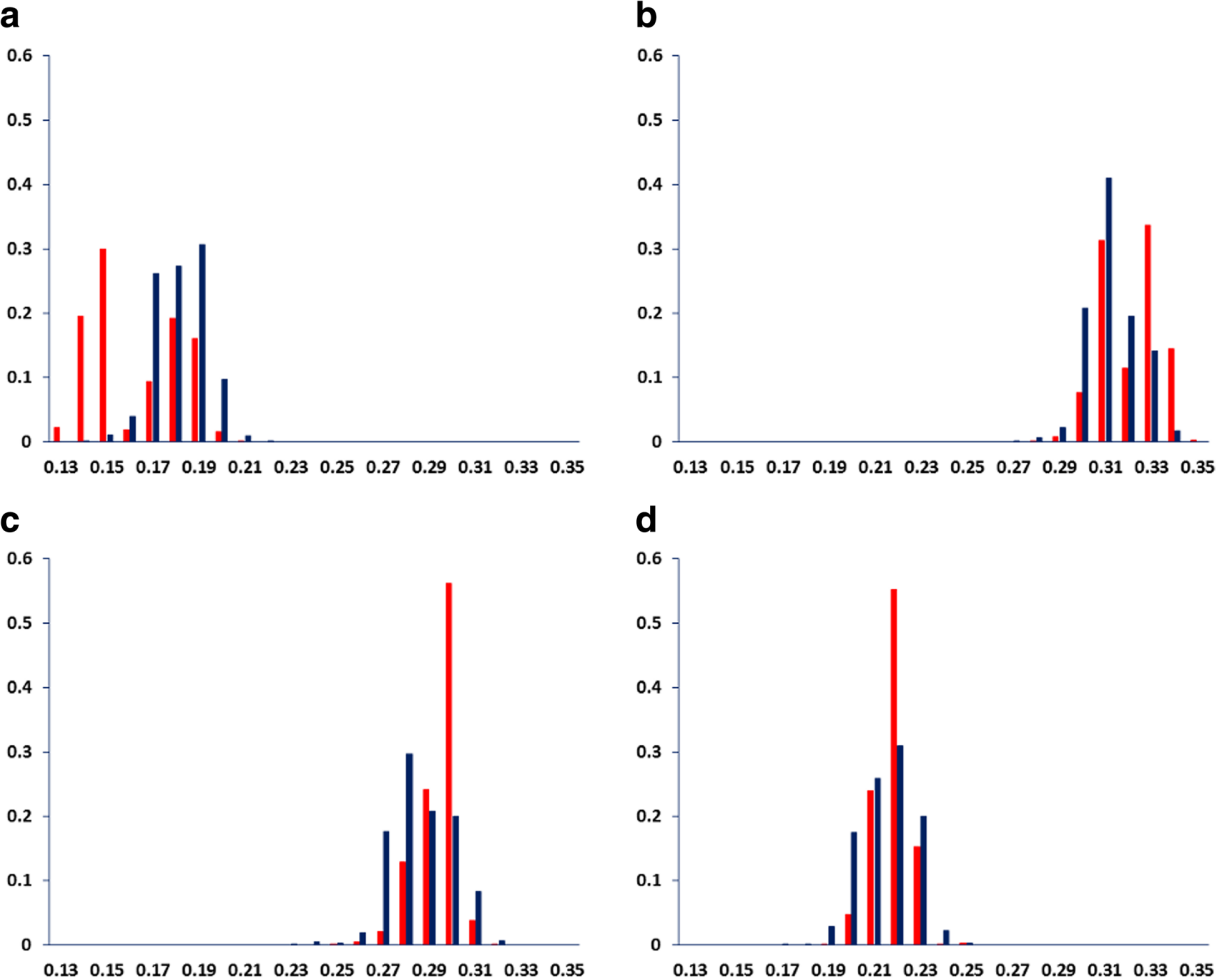
Distribution of HVR1 variants by DNA nt frequency. Shown are binned plots of 5681 HVR1 PhyChem variants derived from 222 patients. Y-axis denote fraction (percent) of variants with same occurrence frequency (x-axis) for: (**a**) nt A, (**b**) nt G, (**c**) nt C and (**d**) nt T. R- and C-associated variants are denoted in red and blue bars, respectively

The Welch’s t-test was used to examine variances in nt and DAC-based PhyChem features in HVR1 sequence data obtained from R (*n* = 124) and C (*n* = 98) patients. Small (range of mean differences: 0.003–0.019) but significant (*p* < 2.20X10^−16^) variance in nt frequencies was observed between the R- and C-associated HVR1 sequence variants (Table 1). In addition, the four DNA nt bases were found to have small but significant (*p* < 2.2 × 10^−16^) correlation to R/C classes by Pearson’s product-moment correlation tests (Table 1). Differences in frequency distributions of DNA nt’s in HVR1 sequence variants from R/C patients are shown in Fig. 1.

**Table 1:** Differences in the population means of DNA nt and PhyChem features of HVR1 and correlation to the R/C classes^§^

Features^a^	t-value (*p*-value)^b^	Means in R/C	Difference in means (95% C.I.)	R-value (95% C.I.)
Nt A	47.86 (<2.20X10^−16^)	0.162/0.181	0.019 (0.019, 0.020)	0.497 (0.477, 0.516)
Nt G	28.64 (<2.20X10^−16^)	0.322/0.313	0.009 (0.008, 0.010)	0.346 (0.323, 0.367)
Nt C	24.26 (<2.20X10^−16^)	0.294/0.286	0.008 (0.007, 0.008)	0.332 (0.309, 0.355)
Nt T	9.61 (<2.20X10^−16^)	0.218/0.215	0.003 (0.002, 0.003)	0.138 (0.112, 0.163)
Twist-tilt	43.39 (<2.20X10^−16^)	0.006/−0.010	0.016 (0.015, 0.017)	0.539 (0.520, 0.557)
Slide-rise	42.22 (<2.20X10^−16^)	−0.037/−0.058	0.021 (0.020, 0.022)	0.500 (0.480, 0.519)
Enthalpy	41.01 (<2.20X10^−16^)	−0.206/−0.250	0.044 (0.041, 0.045)	0.497 (0.477, 0.516)
Breslauer-dH	41.01 (<2.20X10^−16^)	−0.184/−0.231	0.047 (0.044, 0.048)	0.494 (0.474, 0.513)
Breslauer-dG	40.17 (<2.20X10^−16^)	−0.298/−0.273	0.025 (0.024, 0.026)	0.477 (0.457, 0.497)
Protein-DNA twist	37.96 (<2.20X10^−16^)	−0.326/−0.381	0.055 (0.051, 0.057)	0.472 (0.451, 0.492)
Slide-2	36.90 (<2.20X10^−16^)	−0.380/−0.448	0.068 (0.064, 0.072)	0.471 (0.450, 0.490)
SE-ZDNA^c^	36.46 (<2.20X10^−16^)	−0.264/−0.313	0.049 (0.045, 0.050)	0.468 (0.447, 0.488)
Twist-1	36.91 (<2.20X10^−16^)	−0.302/−0.358	0.056 (0.052, 0.058)	0.462 (0.442, 0.483)
G-content	37.79 (<2.20X10^−16^)	−0.375/−0.434	0.059 (0.056, 0.062)	0.457 (0.436, 0.477)
Helix coil transition	34.05 (<2.20X10^−16^)	−0.285/−0.350	0.065 (0.062, 0.070)	0.455 (0.434, 0.475)
MGD^d^	35.72 (<2.20X10^−16^)	0.321/0.353	0.032 (0.030, 0.033)	0.454 (0.433, 0.475)
Sugimoto_dG	37.43 (<2.20X10^−16^)	0.502/0.462	0.040 (0.037, 0.042)	0.450 (0.429, 0.470)
Sugimoto_dS	38.17 (<2.20X10^−16^)	0.520/0.475	0.045 (0.043, 0.048)	0.450 (0.429, 0.471)
Propeller twist	34.58 (<2.20X10^−16^)	0.196/0.148	0.048 (0.045, 0.050)	0.448 (0.427, 0.469)

^a^the four DNA-specific nt’s and the 15 DNA-specific PhyChem properties of HVR1 sequences with R-values ≥0.5 are shown. Detailed description of the DNA PhyChem features used herein is available in [6, 7]

^b^ *p*-value is the same for the Welch two sample t-test and Pearson’s product-moment correlation test

^c^abbreviation for: Stabilizing Energy of Z DNA

^d^abbreviation for: Minor Groove Distance

^**§**^R-values, t-values and differences in means are reported as absolute values

**Fig. 2:**
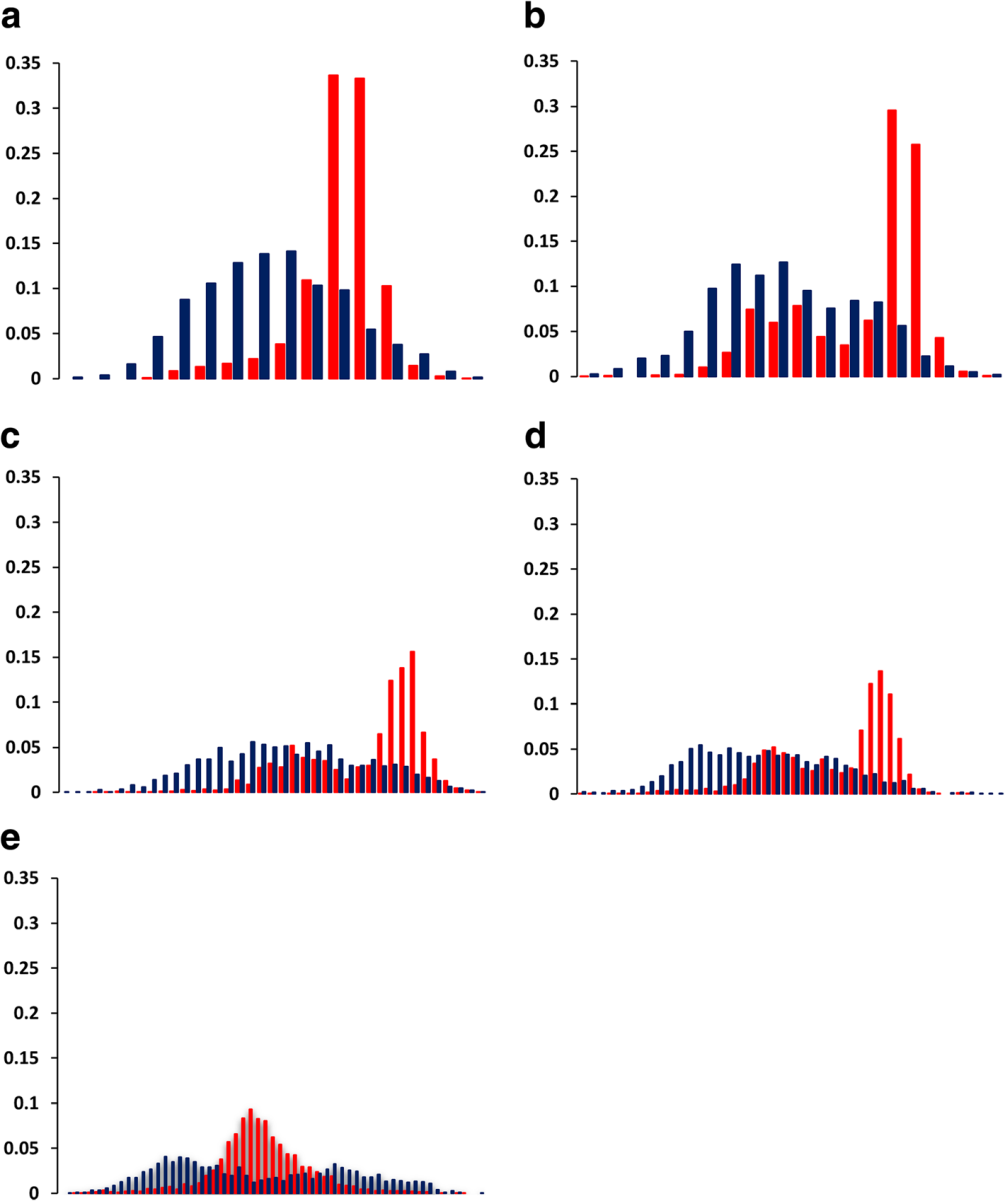
Distribution of HVR1 variants by DNA PhyChem property. Shown are binned plots of 5681 HVR1 PhyChem variants derived from 222 patients. Y-axis denotes fraction (percent) of variants with same range of values (x-axis) for PhyChem indexes: (**a**) Twist_tilt, (**b**) Slide_rise, (**c**) Enthalpy, (**d**) Breslauer_dH and (**e**) Sugimoto_dH. The Sugimoto_dH index illustrates an example of a DNA PhyChem property found to have small but significant correlation (*r* = 0.102; *p* < 1.38 × 10^−14^) to the R/C classes. R- and C-associated variants are denoted in red and blue, respectively

**Fig. 3:**
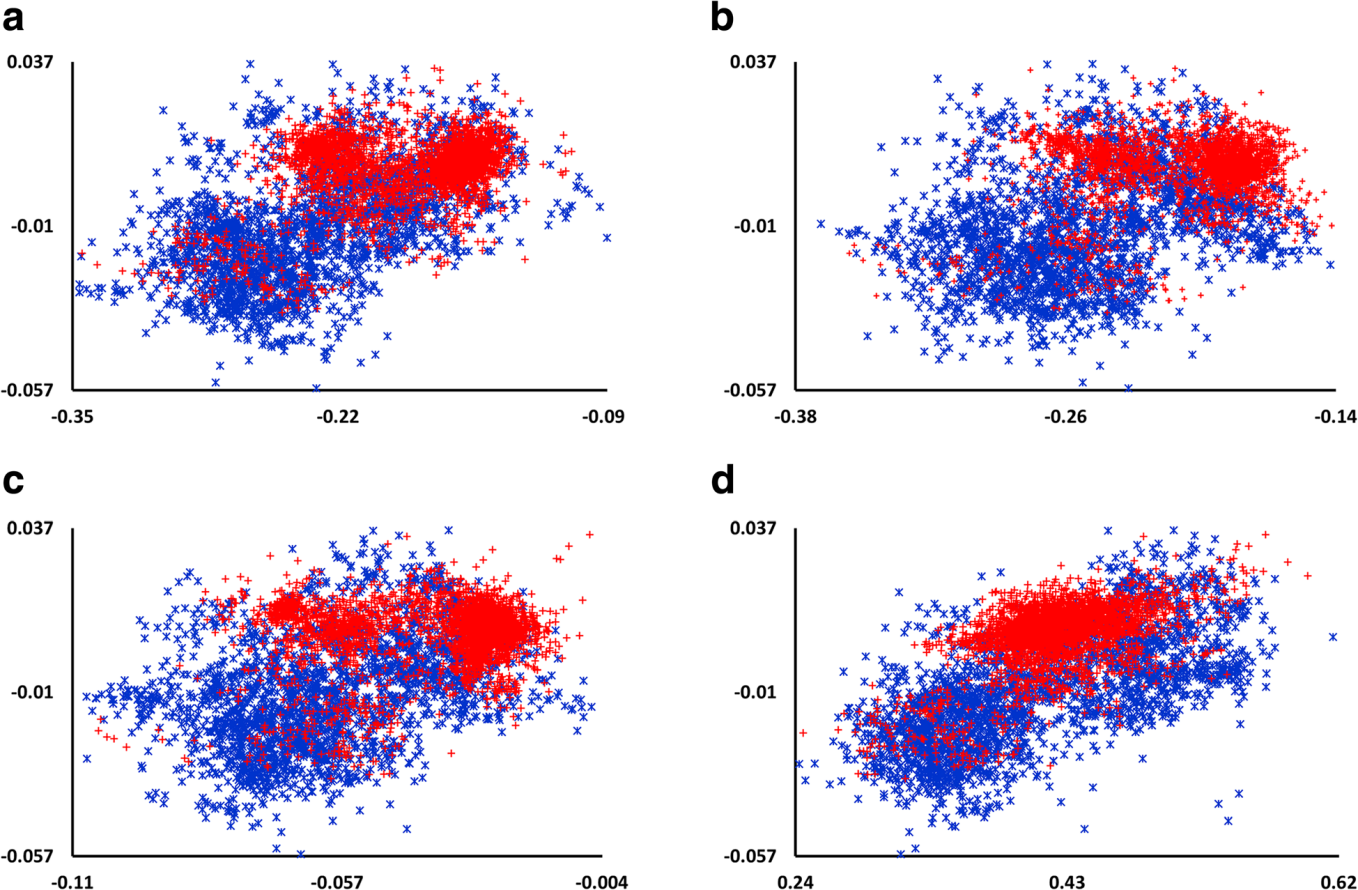
Distribution of HVR1 variants in pairwise DNA PhyChem property plots. Shown are two-dimensional (2D) plots of 5681 HVR1 PhyChem variants derived from 222 patients. The x-axis represents the range of values of the PhyChem indices: (**a**) Breslauer_dH; (**b**) Enthalpy; (**c**) Slide_rise, and (**d**) Sugimoto_dH. Y-axis denotes range of values for the Twist-tilt PhyChem index. R- and C-associated variants are denoted in red and blue, respectively

With exception of three PhyChem features, Welch’s t-test produced values that fell inside the 95% confidence interval (C.I.) and t-values > 3.30 for the remaining 145 PhyChem indexes of DNA dimers used to represent PhyChem variants of HVR1. Differences in the means of such indexes between the R- and C-associated HVR1 PhyChem variants (range of mean differences: 0.003 to 0.068) were found statistically significant (*p*-values ranging from < 9.58 × 10^−4^ to < 2.2 × 10^−16^). Differential distribution of the R/C-associated HVR1 PhyChem variants was observed in equal-width binning plots (Fig. 2) and pairwise scatter plots (Fig. 3).

Among all tested, 145 DNA PhyChem features of HVR1 were found to have small-to-medium correlation with the R/C classes at the statistical significance level of *p* ≤ 0.001, of which 104 features performed similar to nt bases in terms of the degree of correlation (range of R-values: 0.137–0.539) and statistical significance (*p* < 2.2 × 10^−16^). The HVR1 DNA PhyChem features (*n* = 15) with statistically significant (*p* < 2.2 × 10^−16^) medium correlation (R-values ≥ 0.5) to R/C classes are shown in Table 1. Evaluation of the feature-class relationship of several feature subsets (*n* = 10,927) by a merit scoring method [13] showed that a relevant association (*Merit* ≤ 0.416) to the R/C classes could be observed for feature subsets comprised of only 22 DNA PhyChem features of HVR1. Moreover, such feature-class associations were not found in the randomly-labeled datasets (*Merit* = 0 in 37,000 evaluated feature subsets/per random dataset).

Similar analyses on the HVR1 QS data from 25 C- and 54 R-patients indicated no major differences between the training/test data in terms of the minimum/maximum range of values for the 148 DNA PhyChem features investigated here, as well as in terms of the differential R/C-association in the distributions of the PhyChem variants in binned plots (data not shown).

### Spatial distribution of HVR1 PhyChem variants from R/C patients

**Fig. 4:**
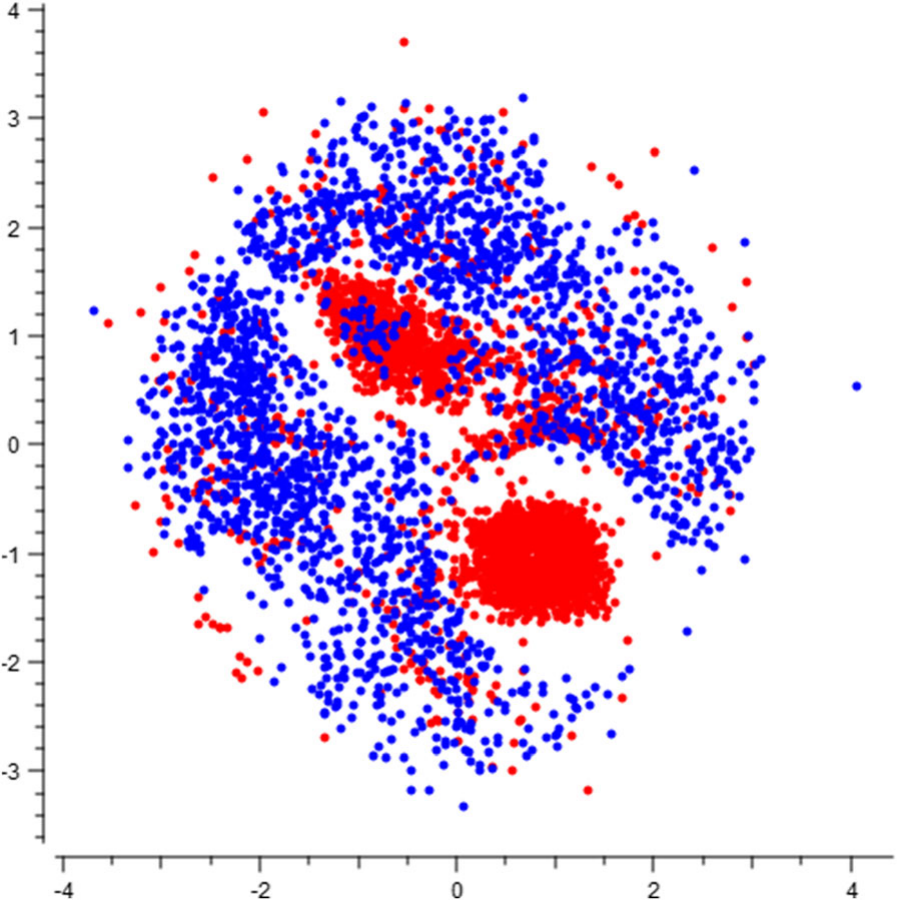
Spatial distribution of HVR1 variants in a 2D MDS plot. Sammon mapping of 5681 HVR1 PhyChem variants derived from 222 patients. MDS plot with average stress = 0.386385 after 224 iterations. R- and C-associated variants shown in red and blue points, respectively

Differential distribution of various PhyChem properties for the R- and C-HVR1 variants (Figs. 2 and 3) suggests association between the HVR1 PhyChem structure and duration of HCV infection. The R-variants have a less uniform distribution of properties, indicating the existence of preferred PhyChem states for HVR1 variants detected during recent infection. A non-linear unsupervised mapping method was used to examine the PhyChem structure of HVR1 data sampled from R (*n* = 124) and C (*n* = 98) patients. In a MDS plot, the R-associated PhyChem variants of HVR1 were observed to occupy a more central and restricted PhyChem space than the C-associated variants, which displayed a much broader distribution (Fig. 4). Such differences in spatial distribution between the R/C-HVR1 PhyChem variants suggest applicability of the properties for developing computational models to discriminate between the R/C-states of infection.

### Classification tests

The RBFNN machine-learning technique was applied to the data representation of DNA PhyChem variants to generate a classifier for identification of R- and C-associated HVR1 variants. Classification performance evaluation of the RBFNN classifier indicates a high accuracy in identification of R- and C-associated HVR1 PhyChem variants in 10xCV tests (Table 2). The individual R/C-variants in the testing dataset were classified with ~84% accuracy whereas the randomly-labeled testing and training datasets were classified at a significantly lower accuracy level (AUROC = 0.5) of ~40% and 60%, respectively. The model was applied to classification of patients using a majority vote rule when the duration of infection is defined by the R/C-class comprising > 50% of all intra-host HVR1 variants sampled from the patient. Duration of infection was classified with accuracy of 88.0% for C-patients and 88.89% for R-patients, intra-host HVR1 variants of which were used in the testing dataset, with the overall classification accuracy being 88.45%.

**Table 2:** RBFNN performance in R/C classification of Intra-host HVR1 PhyChem variants^a^

Dataset	CA	F_1_ measure	MCC	AUROC
Full train set^b^	95.795%	0.958	0.910	0.986
Train set	94.847%^c^	0.948^c^	0.890^c^	0.979^c^
Test set	84.145%^d^	0.842^d^	0.670^d^	0.912^d^
Random-labeled train set	59.038%^e^ (±1.28)	0.521^e^ (±0.007)	−0.007^e^ (±0.022)	0.501^e^ (±0.012)
Test set	39.965%^f^ (±1.948)	0.280^f^ (±0.070)	0.003^f^ (±0.145)	0.385^f^ (±0.144)

^a^For description of train/test data, see Methods Section

^b^Values obtained from RBFNN classifier trained on entire training dataset without CV

^c^Overall value represents averaged values of 10xCV data

^d^Value obtained from RBFNN classifier trained on training dataset by 10xCV

^e^Overall value represents averaged values of 10xCV data obtained from 4 datasets. Standard deviation (*SD*), in parenthesis

^f^Overall value represents averaged values obtained from 4 RBFNN classifiers trained on randomly-labeled data by 10xCV (*SD*)

In addition, the RBFNN classifier exhibited a near identical classification performance on three randomized training datasets and showed no significant (*p* < 0.001) differences with the training set used to initialize the RBFNN classifier prior to validation with the test dataset (Table 3). Such observation, taken together with small variations in performance between the full training and the 10xCV training (Table 2), indicate robustness of the RBFNN classifier.

**Table 3:** Comparison of RBFNN performance on randomized datasets in 100 10xCV tests^§^

Dataset	No. CV runs	CA	F_1_ measure	MCC	AUROC
Train set 1^a^	1000	94.943% (±1.067)	0.960 (±0.009)	0.892 (±0.023)	0.981 (±0.005)
Train set 2	1000	95.958% (±0.717)	0.974(±0.005)	0.887 (±0.020)	0.986 (±0.003)
Train set 3	1000	96.014% (±0.719)	0.974 (±0.005)	0.889 (±0.020)	0.987 (±0.003)
Train set 4	1000	95.981% (±0.699)	0.974 (±0.005)	0.889 (±0.019)	0.986 (±0.003)

^§^Comparisons are based on the corrected two-tailed T-test at a significance level of *p* < 0.001

^a^Dataset used to train (fit) the RBFNN classifier (1st and 2nd rows in Table 2)

## Discussion

Here, we explored a data transformation approach based on DAC of 148 DNA PhyChem properties for identification of association between intra-host HVR1 variants and duration of HCV infection. HCV is an RNA virus. Considering a limited availability of RNA PhyChem properties, we used a DNA-specific representation, which may not be entirely accurate when applied to RNA. However, the classification accuracy achieved here testifies to applicability of DNA PhyChem properties, at least those selected in this study, to the detection of R/C-state of HCV infection. The framework applied here is readily extendable to using RNA DAC features. It can only be expected that performance of the model will improve when the corresponding RNA PhyChem property data become available.

Welch’s t-test indicated that the nt frequency and PhyChem property distributions along the HVR1 genomic region significantly (*p* < 0.001) differ between the R- and C-associated HCV strains examined in this study. However, differences between the R/C HVR1 variants became markedly appreciable after applying the DNA DAC transformation method to the NGS data (Table 1 and Figs. 1 and 2). Furthermore, the finding that 70.3% of the PhyChem features had a significant (*p* < 2.20X10^−16^) correlation to R/C, with R-values ranging between 0.137–0.539, represents a 96.2% increase of correlative features over the original 4 nt-based information (range of R-values 0.138–0.497). In addition, in binned plots (Figs. 1 and 2), the difference between the R/C-associated variants was notably greater for PhyChem representation. Taken together with the Merit scores observed for feature subsets, findings suggest that: (*i*) the DNA DAC-based features provide a better discrimination for differentiation between the R/C classes than the nt diversity alone; and (*ii*) more importantly, there are substantial differences in the PhyChem structure between HVR1 variants from the R and C classes.

Association between the PhyChem structure of HVR1 variants and the R/C classes is complex. The data indicate that, although HVR1 variants from both classes are intermixed in all plots (Figs. 2, 3 and 4), majority of the R-variants appear to cluster. This observation indicates that the R-HVR1 variants have preferred PhyChem properties and majority of them constitute only a fraction of the entire PhyChem space occupied by C-HVR1 variants. Thus, the dominant HCV population established during the early stage of infection has HVR1 variants with certain PhyChem properties and evolves during infection into a population containing HVR1 with a wide range of the properties. Frequent establishment of dominant populations early during infection in recipients from minority HCV variants transmitted from the source cases during outbreaks [15] is in concert with this supposition.

Differences in PhyChem properties between HVR1 from R/C classes are substantial. Although the identified here associations may be affected by variation in sampling of intra-host HVR1 variants, the data indicate that the duration of HCV infection is reflected in evolution of HVR1 through the PhyChem space in each infected host. Performance of the RBFNN classifier on the randomized training datasets (Table 2) and on the randomly-labeled dataset (Table 1), in conjunction with performance on the test dataset (Table 1), suggest that association to R/C is likely due to specific HVR1 traits rather than to the biased sample selection or existence of random statistical correlations in the data. This conclusion is in concordance with prior observations. Previously, we showed that the intra-host HVR1 evolution is associated with the R/C-states of HCV infection [4] as well as with age, gender and ethnicity of hosts and response to interferon treatment [16, 17]. The product of HVR1 expression belongs to a class of proteins known as intrinsically disordered proteins (IDPs) or regions (IDPR) [18, 19]. In general, IDPs/IDPRs have been strongly associated with a multitude of biological functions [20] and play a significant role in evolution [21]. Thus, it seems reasonable to suggest that HVR1, as IDPR, actively participates in the intra-host HCV adaptation and plays specific roles at different stages of HCV infections. The HVR1 functions are likely reflected in changing genetic composition, which is detected using the model developed in this study.

The classification accuracy of the RBFNN classifier (Tables 1 & 2) indicates that the features representing the PhyChem structure of HVR1 can serve as reliable biomarkers of the R/C-association. Based on our findings, we propose that the DNA-specific formulation used herein for the PhyChem representation provides general, information-rich features for detection of trait-specific HVR1 associations beyond the R/C-states of HCV infection, and is potentially applicable to any genomic region. Continued research of such types of features may contribute further to improvement of computational models for the detection of various biological and epidemiological traits from genetic data.

## Conclusions and future work

The HVR1 NGS data contain genetic information, which is pertinent for the identification of the R/C-state of HCV infection. Clustering of the R-HVR1 variants in the PhyChem space suggests a particular way of the intra-host HCV evolution in the space during infection and offers a new approach to the detection of R/C-infections. Identification of new features, which can be extracted from NGS data directly and without using MSA, and development of the model, which accurately detects duration of HCV infection, paves a way for designing cyber-molecular diagnostics for the identification of traits of clinical and epidemiological relevance using genetic data.

Unlike the laboratory diagnostic methods for identification of acute HCV infection, our approach is based on extracting PhyChem features from NGS data and using an RBFNN classifier for identification of the R/C-infections, and, thus, suitable for being hosted by Global Hepatitis Outbreak and Surveillance Technology (GHOST) – a web-based virtual diagnostic system for extraction of public health information from sequence data (see paper in this issue). In addition, our study highlights the importance of considering genomic regions that encode IDPs or IDPRs as potential sources of predictive biomarkers, as well as relevance of the examination of HVR1 in biomarker discovery projects for detection of HCV-related traits. We are currently expanding investigation into DNA PhyChem features expressing higher tiers of interaction between nt-dimers (i.e., Lag > 1) and finalizing a python-based script, which will be made available to authenticated users of GHOST (https://webappx.cdc.gov/GHOST/) for further testing and validation.
